# Online Yoga Pilot Intervention for Black Women at High Cardiovascular Risk: Internet-Based Recruitment and Engagement

**DOI:** 10.2196/41221

**Published:** 2025-09-17

**Authors:** Candace Crosby Johnson, Pascaline Ezouah

**Affiliations:** 1 Nursing College of Health Sciences Lawrence Technological University Southfield, MI United States; 2 Ezouah Consulting, LLC Atlanta, GA United States

**Keywords:** web-based interventions, digital health, mHealth, self-management, eHealth, health promotion, yoga, mind-body therapies, complementary therapy, African Americans, Blacks, women’s health, metabolic syndrome

## Abstract

**Background:**

Disproportionately adverse heart health outcomes in Black women, characterized by high metabolic syndrome prevalence, underscore the need for innovative, accessible interventions. Digital health strategies, particularly web-based yoga videos, show promise for engaging this high-risk group in health-promoting behaviors.

**Objective:**

This study aimed to evaluate the feasibility and acceptability of a web-based yoga intervention for community-dwelling Black women, providing preliminary data to inform a larger, mixed methods study on reducing cardiometabolic risks.

**Methods:**

In this 4-week pilot study, grounded in Pender’s Health Promotion Model, 28 participants engaged in daily online health education and yoga activities through YouTube videos. Using Fitbit trackers, electronic blood pressure monitors, and web-based logs, the study measured metabolic syndrome risk factors and sedentary behavior. Participant experiences were further explored through postintervention focus groups aiming to contextualize the intervention's impact.

**Results:**

We enrolled 28 women, with a completion rate of 79% (22/28), demonstrating successful recruitment and retention. Participants were an average age of 43.3 years with a mean BMI of 40.9 kg/m^2^, indicating a high-risk group for metabolic syndrome. Engagement with 2 or more intervention components were significantly correlated with study completion (*χ*^2^_1_=7.14, *P*=.008). Specifically, viewing over one-half of the instructional videos (*χ*^2^_1_=4.39, *P*=.04) and daily blood pressure monitoring (*χ*^2^_1_=5.67, *P*=.02) were key to participant adherence. The intervention was well-received, with 95% (19/20) of survey respondents finding it satisfactory and suitable. Technology use was high, with all participants having access to the internet, 96% (27/28) owning smartphones, and 53% (15/28) having a YouTube account prior to the study. Recruitment was effectively conducted online, primarily via Facebook and a university newsletter, each accounting for 39.3% (11/28) of participants. The qualitative focus group data unveiled 4 major themes: (1) accountability, emphasizing the shift toward self-prioritization and collective health responsibility; (2) increased awareness, highlighting enhanced understanding of health behaviors and metabolic syndrome risks; (3) health benefits, noting observed improvements in blood pressure and stress levels; and (4) unanticipated stressors, identifying external factors that challenged engagement. These insights underscore the intervention’s multifaceted impact, from fostering health awareness to navigating external stressors.

**Conclusions:**

This pilot study demonstrated the feasibility and acceptability of a culturally tailored, online yoga intervention among community-based, Black women at high risk for metabolic syndrome, showing promising engagement and potential health benefits. The high rates of participation and completion highlight the intervention’s acceptability and the potential for digital platforms to facilitate health behavior changes in high-risk populations. The qualitative findings reveal critical insights into the psychological and social dynamics influencing health behavior change, suggesting the importance of addressing both individual and communal barriers to improve intervention efficacy. Future research should further explore these dynamics in larger, more diverse cohorts to substantiate the intervention’s potential in reducing cardiometabolic risks.

## Introduction

### Metabolic Syndrome

Metabolic syndrome is a predisease classification for individuals who may have a combination of factors that predispose them to diabetes, hypertension, and cardiovascular disease, including sedentary lifestyle, high-stress work and family dynamics, and dietary patterns [1]. Metabolic syndrome risk factors include elevations in the following biophysical markers: blood pressure, fasting blood glucose, cholesterol, triglycerides, and waist circumference. Metabolic syndrome is associated with a 5-fold increased risk for type 2 diabetes, a chronic condition linked to cardiovascular disease, the leading cause of death in US adults [2]. Among US racial/ethnic female subgroups, prediabetes and hypertension are highest in Black women, 40% of whom experience metabolic syndrome. Overall, cardiovascular disease morbidity and mortality are disproportionately highest in Black women when compared with other US subgroups [3].

To manage the risk presented by metabolic syndrome, it is recommended that optimal BMI, blood pressure, and blood glucose levels be maintained through lifestyle approaches such as increasing physical activity and exercise [4,5]. Black women experience comparably high obesity and physical inactivity rates when compared with other US subgroups [6]. Notwithstanding, Black women have difficulties implementing physical activity regimens due to lack of awareness and training, which influence their behavior management strategies [7].

### Multiple Risk Factor Interventions

Nonpharmacological behavioral modification interventions reduce the burden of chronic diseases associated with metabolic syndrome. Research demonstrates that multiple risk factor interventions—programs that impact more than one symptom of metabolic syndrome—can lower blood pressure levels, BMI, and waist circumference in high-risk populations [8]. Behavioral interventions that address lifestyle modifications are recommended in Black patients with slightly elevated blood pressures as they can prevent or delay worsening disease [9]. When developing behavioral interventions, content should include caveats for stress management to help individuals stay engaged with lifestyle modifications [10]. Additionally, incorporating behavior change techniques such as self-monitoring and eliciting social support for exercise further aid with maintaining lifestyle behavioral changes [11]. Behavioral interventions incorporating culturally adapted, participant-involved strategies have been effective over usual care in weight management studies involving Black women [12].

Mind-body therapies that use spinal manipulation such as yoga have been shown to be efficacious in lowering blood pressure and improving lipid profiles, yet to date, there are a shortage of such interventions that reduce metabolic syndrome risk factors in high-risk Black populations [13]. Research suggests mind-body therapies such as mindfulness and yoga offer promise as a therapeutic treatment for cardiovascular disease risk [14]. Mindfulness meditation, a mental component of yoga practice, may improve health outcomes in Black communities. Mind-body therapies are feasible and acceptable interventions that result in positive health outcomes among Black individuals, particularly when cultural adaptations are applied [15]. Remotely delivered yoga interventions including those with postures, breathing techniques, and meditation exercises were shown to be generally safe and feasible for implementation in diverse populations with and without chronic conditions [16].

Novel interactive eHealth—digital health—educational approaches are being explored for patient empowerment and self-management of chronic diseases as they provide comprehensive, accessible content [17]. Remote, web-based interventions have demonstrated evidence for a positive, moderate effect on physical activity [18], showing promise for improving self-management of obesity, a major contributor to metabolic syndrome. Research using mobile devices suggests that this method is effective for influencing physical activity behaviors [19]. Mobile devices can be used to send information to users, gather information from users, and foster peer-to-peer connections [20]. Black women are more likely to be smartphone-dependent and consume more mobile- and internet-based health information than other US ethnic/racial counterparts [21]. When it comes to tracking their health, Black women are no more likely than other racial/ethnic subgroups to use their mobile devices to track their weight, diet, or exercise; however, the likelihood of engaging with eHealth strategies increases when they have one or more chronic conditions or have gone through a significant health change in the past year [21].

### Challenges in Addressing Metabolic Syndrome in Black Women

Despite the efficacy of mind-body therapies like yoga in managing cardiovascular risk factors, there is a noted shortage of interventions specifically designed to reduce metabolic syndrome risk factors among high-risk Black populations. This gap highlights the need for culturally tailored interventions that are both acceptable and feasible for Black women, whose engagement with digital health strategies is often contingent upon existing chronic conditions or significant health changes.

This study aimed to assess the feasibility, engagement, and preliminary impacts of a culturally tailored, online yoga intervention, YogicDance, on metabolic syndrome risk factors and related psychological health outcomes in Black women aged 35 years to 50 years at high cardiovascular risk, addressing the unique needs and barriers faced by this population while evaluating its effectiveness at improving both metabolic syndrome risk factors and psychological health outcomes. Specifically, it evaluates participant enrollment and participation rates, their interaction with the intervention’s technological components, and their perceptions of their satisfaction with the intervention as well as its suitability, ease of use, and helpfulness.

## Methods

### Study Design

**Figure 1:**
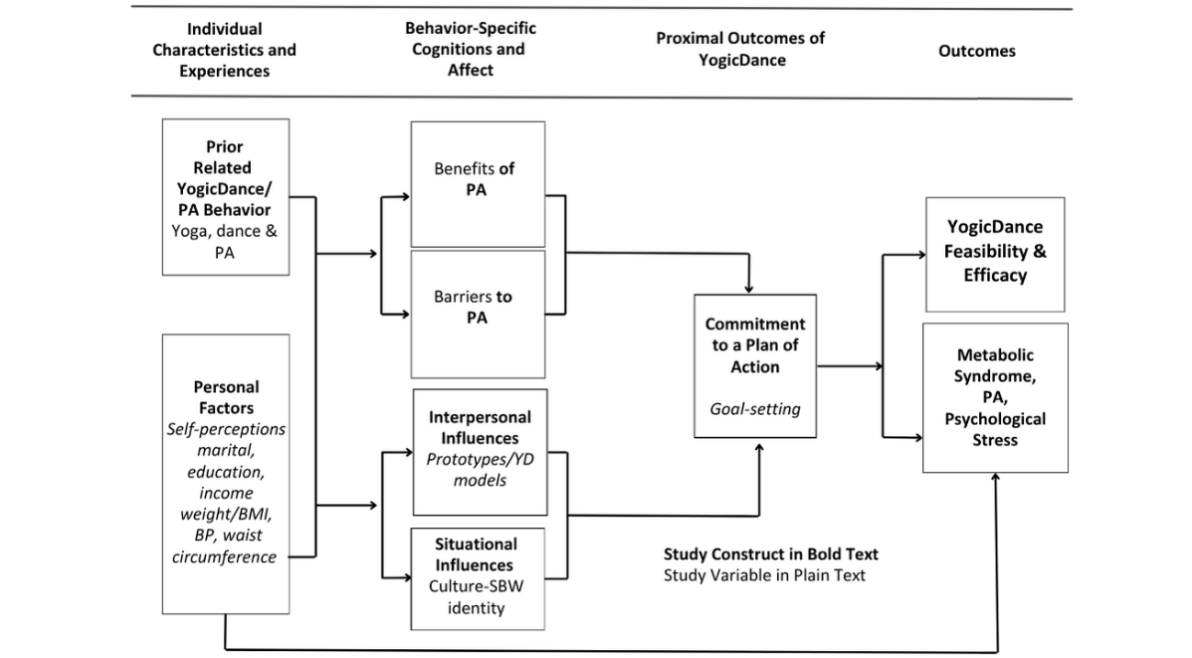
Health Promotion Model adapted for the YogicDance (YD) intervention study. PA: physical activity; BP: blood pressure; SBW: strong Black woman.

The Health Promotion Model (HPM), outlined in Figure 1, is the framework that guided the foundational constructs to be explored in this pilot study. The HPM combines concepts from social cognitive theory [22] and expectancy-value theory [23] to facilitate researchers’ understanding of the “background factors” [24] that influence health-promoting behaviors. The HPM has been used to explore the impact of health promotion strategies on physical activity [25], in older women [24], in Black women [26] and in Black women at risk for chronic disease [27]. The HPM elicits input from the participants regarding their individual characteristics and experiences as well as behavior-specific cognitions and affect including benefits and barriers related to intervention engagement. This culturally tailored physical activity and stress management intervention sought to address the complexities of the Black female experience with regards to accessing health information and engaging in health behaviors that ultimately reduce poor clinical health outcomes. The study was designed with selected ecological factors such as low access to physical activity resources, unsafe neighborhood environments, and cultural sensitivity in mind.

**Figure 2:**
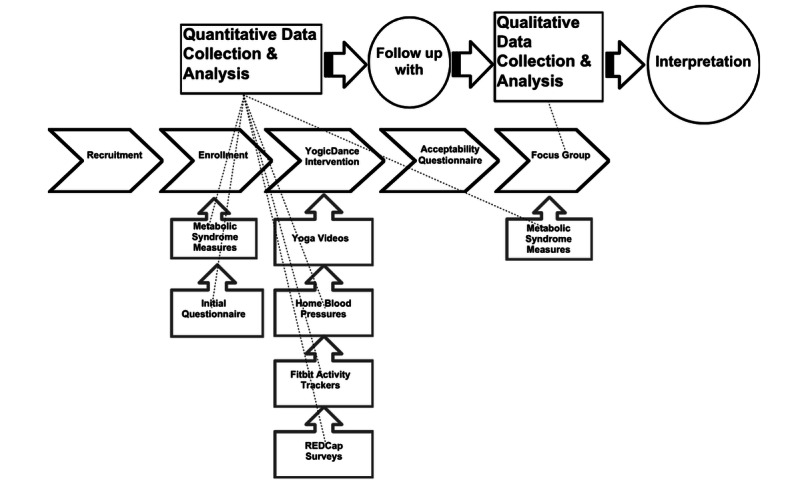
YogicDance explanatory sequential study design. REDCap: Research Electronic Data Capture.

A mixed methods approach was selected to capture the participants’ holistic perspectives on the acceptability of the YogicDance intervention. This study used an explanatory sequential mixed methods design [28]. Initially, quantitative data were collected and analyzed to measure the metabolic syndrome risk factors and physical activity levels of the participants using tools such as Fitbit trackers and electronic blood pressure monitors. Following this, qualitative data were gathered through focus groups to explore participants’ experiences, barriers, and benefits related to the intervention. The qualitative findings were used to provide deeper insights and context to the quantitative results, allowing for a comprehensive understanding of the intervention’s impact. This approach enabled the researchers to integrate quantitative outcomes with qualitative insights, thereby enhancing the overall interpretation of the study findings. A mixed methods approach was implemented because the complex determinants of physical activity in Black women warrant corroboration to give researchers deeper, contextual meaning to the broad, descriptive correlates assessed in this study. Focus group data provided contextual understanding of the relationships among study variables found through quantitative data measures [29]. A joint visual display that integrates the collection and analysis of both the quantitative and qualitative data was used to help enhance the clarity of the data integration in the final interpretation (see Figure 2).

### Eligibility Criteria

Eligibility screenings took place initially by telephone or in-person meetings in settings acceptable to and agreed upon by the interested individual and the research team. The inclusion criteria for the YogicDance study were as follows: Black woman (self-identified volunteers), aged 35 years through 50 years (valid ID); overweight (BMI >25 kg/m^2^), abdominally obese (waist circumference >88 cm), available at-home internet and desktop or laptop screen access (research team observation), email access (confirmation email), able to navigate the YogicDance website (per YouTube video link access), and able to increase physical activity (per a “no” response to all questions on the Physical Activity Readiness Questionnaire+ [PAR-Q+]) [30]. Additionally, a health care provider’s clearance was required for interested women to proceed further with the study if they were extremely obese (BMI >40 kg/m^2^); had blood pressure measures above 140 mm Hg (systolic blood pressure) or 90 mm Hg (diastolic blood pressure); answered “yes” to one or more of the 7 PAR-Q+ questions; or reported taking medications or being under a health care provider’s care for a diagnosis of diabetes, hypertension, or any other health condition.

The age range of 35 years through 50 years was chosen because this is an age group at high risk for the indicators seen in metabolic syndrome but who may not yet have exhibited cardiovascular disease, thus allowing an opportunity for prevention of disease. Individuals in this age range also tend to have busy lives with competing family and work responsibilities that contribute to identification with stress and sedentary lifestyles. YogicDance integrated the aforementioned strategies as YouTube-based eHealth education videos, cultural tailoring using Black models or educators, self-monitoring via commercially available activity trackers, and stress management using yogic breathing and movement techniques. Interested women were excluded from participating in the study if they were currently undergoing physical therapy, had symptoms of an untreated musculoskeletal injury, were housebound because of physical disability, walked with assistance or assistive devices, or were unable to secure a health care provider’s clearance to participate in the YogicDance study. Interested individuals who met all the inclusion criteria (as observed or assessed by the study team) were consented into the study after having the consent form explained to them and exhaustively responding to questions about study participation. Screened women who met all of the initial inclusion criteria were scheduled for an in-home visit. During the (in-home) baseline data collection, the study team also confirmed that the potential participant had an internet connection and access to a working smartphone. In addition, the team observed that the individual had sufficient space to carry out the YogicDance movements with a clear view of a computer screen.

### Recruitment

Participants were recruited using a variety of methods including printed institutional review board (IRB)–approved fliers, university campus TV monitors, and online newsletter advertisements containing research team contact information and depicting relevant images of Black women of varied body sizes. Study personnel made appearances at community health fairs and at hair salons to recruit Black women. Given the nature of this internet-based intervention, digital versions of the IRB fliers were also distributed on internet-based marketplaces such as Facebook and YouTube (see Multimedia Appendix 1). Because Blacks underparticipate in clinical trials, recruitment and attrition are significant problems for researchers. Although using a small sample size carries the risk of making a type II error, a sample of 30 participants was deemed appropriate to detect intervention effects. As approximately 40% of Black adult women have metabolic syndrome, we screened 51 individuals to reach our target enrollment of 30 at-risk participants.

### Intervention Details

**Figure 3:**
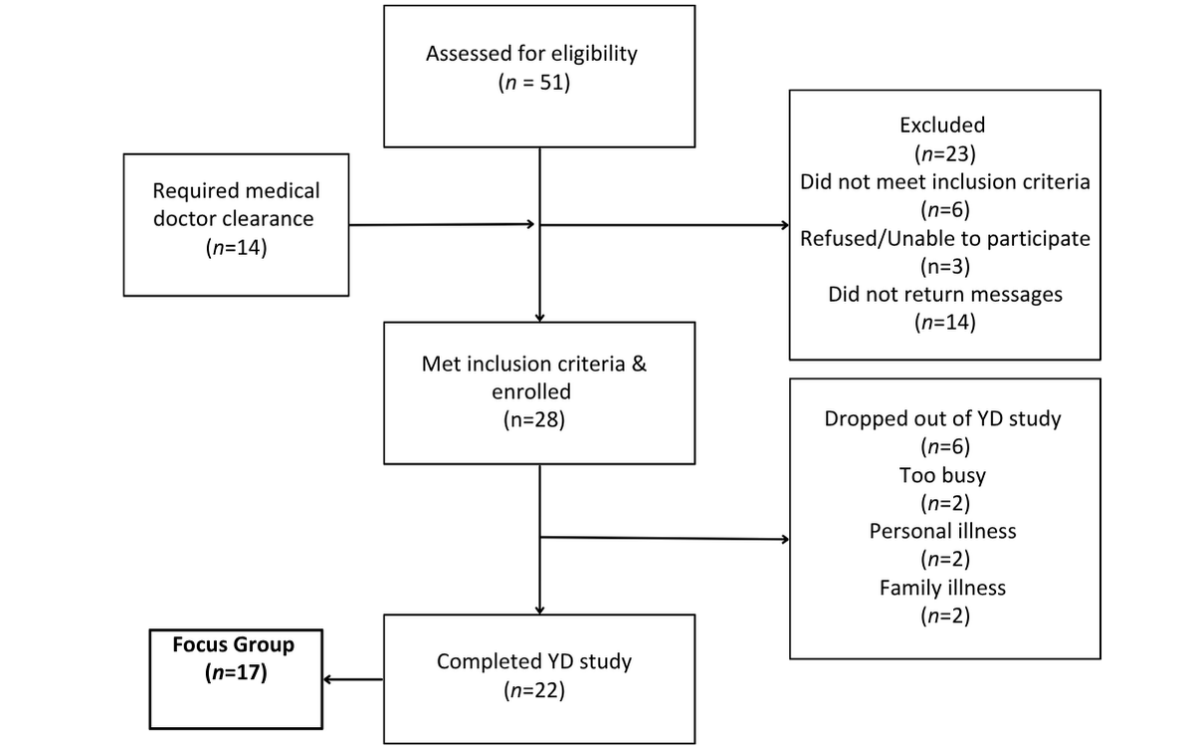
YogicDance (YD) participant flow diagram.

Study recruitment and enrollment occurred on a rolling basis until the target enrollment was reached (see Figure 3 and Multimedia Appendix 2). The focus groups were held in 2 trusted, community-based locations on and around the university campus. Focus groups were used to assess the feasibility of the intervention, explore barriers and benefits to participating in YogicDance, and explore the contextual meaning and experiences with yoga in the lives of the participants during the study. Each participant was given two US $25 gift cards (at pre- and postintervention) as remuneration for their time and study involvement.

Enrolled study participants were assigned a Fitbit (Fitbit Inc) activity tracker and Omron (Omron Corporation) blood pressure monitor and were assisted by the research team in calibrating these monitoring devices to be used in the study. Immediately following calibration, the team assisted the participant with installing the device software and downloading the companion apps from Fitbit, Omron, and YouTube. To protect the participants’ confidentiality, each participant was also given a card with passwords to the aforementioned website accounts, and assigned identification numbers were used to protect the participants’ privacy. Baseline physical activity status (sedentary vs physically active) was determined using the International Physical Activity Questionnaire (IPAQ). To determine baseline metabolic syndrome risk, the research team collected metabolic syndrome–related anthropometric (weight and waist circumference), physiologic (systolic and diastolic blood pressures), and fasting blood (glucose and lipid profile) measures. Participants were also instructed to watch a series of introductory, safety-related, health education and goal-setting videos prior to beginning YogicDance physical activities. Additionally, participants were asked not to alter their diets or other health-related activities to reduce study intervention confounders.

During the 4 weeks of the intervention, participants watched YogicDance physical training videos and performed 10 minutes to 30 minutes of physical activity with their choice of 3 video training modules available on the YouTube website. YogicDance instructors included 2 women. The first was a registered nurse and yoga instructor (White American, 64 years old) with 500 hours of yoga training and additional certifications in breath-centered yoga as well as yoga for cardiovascular disorders, osteoporosis, and arthritis. Her 40 years of yoga instruction included experience in teaching yoga to community-based Black women with and at risk for cardiometabolic disease. She developed and instructed the participants in gentle, restorative yogic breathing and postures (asanas) in chair and mat yoga formats. The second was a certified physical education instructor and physical trainer (Black, 42 years old) with 20 years of training experience with Black female clientele who provided education and instructional training in warm-up and cool-down stretching using light resistance bands and moderate aerobic activity. All programs began with a warmup and concluded with a cool-down session. YogicDance instructors provided instruction on how to safely execute yoga and resistance training activities, with specific guidance for effective breathing, timing, and body positioning. Immediately following YogicDance practice, participants were asked to measure their blood pressures using the Omron electronic monitor as well as heart rate. Participants were also provided a paper booklet for recording their blood pressures in writing, to help them with recall in the event they are not able to complete online-based surveys administered using the Research Electronic Data Capture (REDCap) platform immediately following YogicDance practice.

Weekly during the intervention, study staff contacted participants by telephone to communicate any questions; concerns; problems; or reports of pain, injury, or adverse events. The research team helped the participant assess their pain or injury, and any participant reporting physical distress or acute symptoms such as intense pain, numbness, tingling, burning, swelling, redness, and tenderness was encouraged to make an appointment with their health care provider. Participants also had the opportunity to contact the research team after business hours and on weekends during the study to report any adverse events, pain, or injury.

### Quantitative Phase

#### Data Collection

##### Baseline Measures

Baseline physical activity status (sedentary vs physically active) was determined using the IPAQ [31]. To determine baseline metabolic syndrome risk, the research team collected metabolic syndrome–related anthropometric (weight and waist circumference), physiologic (systolic and diastolic blood pressures), and fasting blood (glucose and lipid profile) measures. Baseline data collection involved assessing various parameters to understand the participants’ health status. The data collection included several measures and tools, which are described in the following sections.

##### Demographic Characteristics Survey

Age, education level, household income, marital status, and employment status were collected using a standardized questionnaire developed by the principal investigator (PI). The frequencies and durations of walking, moderate activity, and vigorous activity over the past week were assessed using the IPAQ.

##### Participant Prior Technology Use Survey

A standardized, PI-developed questionnaire assessed technology use prior to the intervention as well as internet-based recruitment methods (eg, Facebook, email fliers, University listserv fliers) used to recruit the study participants.

##### YogicDance Study Engagement

Engagement metrics included the total number of study activities completed, videos watched, Fitbit worn, web-based surveys taken, and blood pressures recorded.

##### YogicDance Study Acceptability Survey

This PI-developed survey included participant feedback on the screening process and overall acceptability of the intervention.

#### Data Analysis

Descriptive statistics were used to calculate recruitment, refusal, attrition, and retention rates as well as reasons for refusal. Descriptive statistics were used to describe the sample and YogicDance intervention acceptability, including calculating means, standard deviations, and ranges for the continuous variables and counts with frequencies for the categorical variables. Chi-square analyses were used to explore significant relationships among categorical study engagement variables. These study activities included watching YouTube videos, wearing Fitbit activity trackers, taking home-based blood pressures, and completing web-based activity logs. The levels of significance were set at an α of .05 for all statistical analyses.

Quantitative data were analyzed using SPSS (Version 25; IBM Corp). Although this was a pilot study, examining potential differences to determine if there are trends toward improvements in metabolic syndrome risk factors and physical activity levels was important for determining effect sizes. In addition, means and standard deviations from this study will be used to conduct a more precise power analysis for a future larger randomized controlled trial. Pre-post intervention differences in metabolic syndrome risk factors and physical activity outcomes are reported elsewhere.

### Qualitative Phase

#### Data Collection

The focus group participants were a subset of those who participated in the intervention (see Figure 3). Specifically, 17 of the 22 participants who completed the intervention also participated in the postintervention focus groups. These focus groups aimed to assess the feasibility of the intervention and explore barriers and benefits to participation. Two prescheduled focus groups were held in a cultural center and in a classroom located on the university campus. Bus and parking accommodations were provided, and childcare was offered to participants. We provided emailed weblinks to participants wishing to join the focus groups by Skype or GoogleMeet. An interview guide was used to ensure clarity and to avoid redundancy in the open-ended questions. Although an interview guide was used to elicit behavior-specific cognitions and affect of the participants, the open-ended questions and reflective interviewing skills of the researcher encouraged communication. The researcher was able to further prompt the participants regarding the benefits and barriers to engagement with the YogicDance intervention.

Qualitative focus group results provide a composite picture of the YogicDance intervention experience. Analyses of the interview data revealed that they were commensurate with the behavior-specific cognitions and affect construct of the HPM (Figure 1) as we sought to identify the benefits and barriers to YogicDance study participation. Hierarchical language frequency patterns associated with study engagement and self-monitoring were identified during qualitative analyses of transcript data.

#### Data Analysis

Cognitive and affective dimensions of participant engagement were measured by exploring semistructured focus group transcripts and open-ended acceptability survey responses to identify themes associated with study engagement and completion. Focus group interviews were transcribed and analyzed using NVivo 11 Pro for Windows (QSR International) to identify word frequency and other familial patterns related to benefits and barriers to study engagement. The HPM (Figure 1), by Pender, informed the initial identification of behavior-specific cognitions and affect related to intervention feasibility, namely the benefits and barriers to study participation. Source materials were coded for themes using NVivo queries for searching data and organized into hierarchical nodes that were then visualized into data maps interpreted by researchers. The researchers iteratively discussed and compared the coding of the data with emerging themes. The researchers also used member-checking, in which several participants reviewed the results and validated that our identified themes were representative of the experience of participating in the study.

We used deductive content analysis as outlined by Elo and Kyngäs [32] to analyze the focus group discussions. This choice was informed by our mixed methods design, aiming to integrate qualitative insights with quantitative findings for a comprehensive understanding of physical activity determinants in Black women. For the discussions, 17 participants contributed to 2 focus groups, which were audio-recorded and lasted approximately 60 minutes each. Transcriptions and field notes taken during these sessions underwent a rigorous coding process to identify recurrent themes, which were then categorized and analyzed for frequency. The analysis was conducted independently by 2 researchers, including the principal investigator and a qualitative expert in physical activity among Black women, ensuring trustworthiness and reliability through their iterative discussion and consensus. This collaborative analysis process allowed for the triangulation of themes, enriching our quantitative data with contextual depth and supporting the convergence of findings. Trustworthiness was further enhanced by adhering to established qualitative research guidelines, including credibility, transferability, dependability, and confirmability, through methodical data handling, analysis, and interpretation.

### Ethical Considerations

IRB approval was obtained for study forms and procedures from the Virginia Commonwealth University IRB (number HM20004679). No other approvals were needed for study completion. All participants provided written informed consent. Our study used an open, web-based, e-survey design that received IRB approval, including a thorough informed consent process detailing the survey’s duration, data handling practices, and purpose. To ensure the protection of participant data against unauthorized access, robust data protection measures were implemented. The survey development phase included extensive pretesting to assess both usability and technical functionality, optimizing the participant experience. We actively recruited participants through a multifaceted advertising campaign, leveraging social media, email newsletters, and targeted banner ads, aiming to attract a wide-ranging demographic to our convenience sample.

The survey administration was structured to enhance participant engagement and data integrity, featuring adaptive questioning and item randomization to mitigate response biases. Voluntary participation was encouraged with incentives, and multiple safeguards, including the use of cookies and IP address monitoring, were in place to prevent duplicate submissions. Analytically, we addressed the potential nonrepresentativeness of our sample through statistical corrections, analyzing both complete and partially completed questionnaires to extract comprehensive insights. By following the CHERRIES (Checklist for Reporting Results of Internet E-Surveys) checklist, our e-survey methodology was transparent, secure, and methodologically sound, providing valuable data on YogicDance intervention engagement while ensuring participant privacy and data integrity.

For the focus groups, audio-recordings were made to capture the discussions accurately. These recordings were transcribed verbatim by the research team. No video recordings were made to ensure participant comfort and confidentiality. The audio files and transcripts were stored securely on password-protected computers accessible only to the research team. Physical copies of the transcripts were stored in locked cabinets within the research facility. Access to the audio recordings and transcripts was strictly limited to the principal investigators and authorized research team members involved in the data analysis.

## Results

### Participants Description

We enrolled 28 women (Figure 3) using primarily Facebook (11/28, 39%) and a university-wide, e-newsletter (11/28, 39%), and 22 women (age: mean 43.3, SD 4.69 years) completed the study. Of the 22 women who completed the study, 20 (90%) completed the postintervention acceptability survey. Of the 22 study completers, 17 (77%) participated in the postintervention focus groups. Of the women, 50% (11/22) possessed 3 of the 5 aforementioned metabolic syndrome risk factors, with a mean of 2.5 (SD 1.6) metabolic syndrome risk factors in the 28 enrolled women and a mean of 2.6 (SD 1.6) metabolic syndrome risk factors in the 22 study completers demonstrating similarities between the 2 groups (Table 1). Of the 28 women enrolled, 5 (18%) were physically active, 11 (39%) were married, and 24 (89%) were college-educated. The mean BMI for the enrolled sample was 40.9 (SD 8.7) kg/m^2^. The enrolled women (N=28) identified 13 (SD 7.4) lifestyle stressors at study baseline, while the study completers (n=22) identified 11 (SD 7.2) lifestyle stressors at study baseline. Regarding technology, all 28 participants had in-home access to a desktop, tablet, or laptop computer (Table 2). Of the 28 participants, 27 (96%) had “smart” mobile phones, and 25% (7/28) had Fitbit accounts already established prior to the YogicDance study. Over one-half of the participants (15/28, 53%) had a YouTube account prior to the study.

**Table 1:** Participant characteristics.

Characteristics				Total sample (n=28)		Study completers (n=22)
**Demographics**						
	Age (years), mean (SD)		43.0 (4.5)		43.3 (4.7)	
	BMI (kg/m^2^), mean (SD)		40.9 (8.7)		40.9 (8.6)	
	**Marital status, n (%)**					
		Single, never married	10 (36)		10 (46)	
		Divorced, separated, widowed	5 (18)		3 (14)	
		Married, partnered	11 (39)		9 (41)	
	**Highest level of education, n (%)**					
		Some college/associate’s degree	4 (14)		4 (18)	
		Bachelor’s degree	10 (36)		9 (41)	
		Master’s degree	9 (32)		7 (32)	
		Doctorate	2 (7)		2 (9)	
	**Yearly income range (US $), n (%)**					
		10,000-19,999	1 (4)		1 (5)	
		20,000-29,999	2 (7)		1 (5)	
		30,000-39,999	5 (18)		4 (18)	
		40,000-49,999	6 (21)		5 (23)	
		>50,000	12 (43)		11 (50)	
Number of metabolic syndrome risk factors (from a total of 5), mean (SD)				2.5 (1.1)		2.6 (1.2)
Number of social supports, mean (SD)				1.6 (1.6)		1.7 (0.5)
Number of lifestyle stressors, mean (SD)				13.1 (7.4)		11.4 (7.2)
**Activity Status, n (%)**						
	Sedentary-No		3 (11)		4 (18)	
	Sedentary-Yes		25 (89)		18 (82)	
**Metabolic syndrome, n (%)**						
	Low: <3 metabolic syndrome characteristics		13 (46)		11 (50)	
	High: ≥3 metabolic syndrome characteristics		15 (54)		11 (50)	

**Table 2:** Participant technology access and internet recruitment (N=28).

Variables			Results, n (%)
**Prior technology access**			
	Internet	28 (100)	
	Smartphone	27 (96)	
	Laptop, desktop, tablet	28 (100)	
	DVD player	27 (96)	
	Skype	13 (46)	
	Prior activity tracker	2 (7)	
	Prior online Fitbit account	7 (25)	
	Prior YouTube account	15 (54)	
	Prior in-home BP^a^ monitor	7 (25)	
**Internet recruitment methods**			
	Study Facebook page	11 (39)	
	University Facebook page	0 (0)	
	University online newsletter	11 (39)	
	Gmail flier	3 (11)	
	Google Voice message	1 (4)	
	Print flier	2 (7)	

^a^BP: blood pressure.

### Engagement With the YogicDance Intervention

This pilot study supported the feasibility of a larger study in its ability to identify and recruit eligible subjects. Completing the study was associated with being engaged in 2 or more of the 4 main aspects of the intervention including watching the videos, taking daily blood pressures, completing the web-based surveys, and wearing the Fitbit trackers >3.5 days per week (*χ*^2^_1_=7.14, *P=*.008; Table 3). Watching over one-half of the instructional videos was associated with completing the study (*χ*^2^_1_=4.39, *P=*.04), as was taking at-home blood pressures (*χ*^2^_1_=5.67, *P=*.02). Study completion was not associated with merely completing the online surveys alone (*χ*^2^_1_=3.60, *P=*.06) or wearing the Fitbit tracker only (*χ*^2^_1_=0.392, *P=*.53).

**Table 3:** YogicDance study engagement (n=22).

Study engagement measures	Quantitative results	
	Likelihood-ratio, χ^2^ (*df*)	*P* value
Total study activities completed	7.14 (1)	.008
Videos watched	4.39 (1)	.04
Fitbit worn	0.39 (1)	.53
Web-based surveys taken	3.60 (1)	.058
Blood pressure taken	5.67 (1)	.02

### Feasibility and Acceptability of YogicDance Intervention

Acceptability and ease-of-use of the components of YogicDance intervention were evaluated using survey data. Overall, the study participants who completed postintervention acceptability surveys (n=20) found participating in the YogicDance intervention satisfying (19/20, 95%) and following along with the YogicDance video practice suitable (19/20, 95%; Table 4). Nearly all (19/20, 95%) of the women found the overall screening process suitable. In addition, 70% (14/20) of the participants found study researchers coming into their home to do baseline measurements suitable, while 85% (17/20) of the participants thought visiting the urban university campus for postintervention study measures suitable. Furthermore, 80% (16/20) of the women who completed the acceptability survey thought the web-based survey questions were clear, while 95% (19/20) of them found the survey platform, REDCap, to be easy to use. With regards to self-monitoring devices, 70% (14/20) and 95% (19/20) of the women found the blood pressure monitor and the Fitbit activity tracker, respectively, to be easy to use. Though 80% (16/20) of the women found the YogicDance videos to be useful, only 50% (10/20) of the women found the YouTube website to be easy to use. In focus group discussions, participants shared how the use of Fitbits raised awareness about physical activity levels, sleep patterns, and the impact of exercise on blood pressure. In focus group discussions, participants expressed initial enthusiasm about participating in the yoga study and were eager to try something new and improve their health. Some participants were motivated by personal health concerns or a desire to reduce stress. Participants appreciated the immediate feedback on their health metrics, which motivated some to increase their activity levels. Exemplar quotes that represent sentiments of the collective focus group are provided. Pseudonyms are provided so that readers can understand how representative the qualitative data are.

**Table 4:** YogicDance (YD) study acceptability (n=20).

Acceptability survey measure	Quantitative results, n (%)	Exemplar quotes
Screening process suitable	19 (95)	None
Researchers coming to home suitable	14 (70)	“Having the researchers come to my home was convenient and put me at ease.” [Jasmine]
Poststudy visit to campus was suitable	17 (85)	“Visiting the campus for post-study measures was well-organized and professional.” [Jasmine]
Survey questions clear	16 (80)	“The survey questions were clear and easy to understand.” [Aisha]
Easy-to-use web surveys	19 (95)	“The web surveys were very user-friendly and simple to navigate.” [Jasmine]
Easy-to-use BP^a^ monitor	14 (70)	“The blood pressure monitor was a bit tricky at first, but overall, it was a useful tool.” [Aisha]
Easy-to-use Fitbit tracker	19 (95)	“I really enjoyed using the Fitbit; it encouraged me to be more active.” [Ebony]
YD video practice suitable	19 (95)	“Following along with the YogicDance videos was suitable and enjoyable.” [Aisha]
Helpful health education videos	18 (90)	“The health education videos were very helpful and informative.” [Aisha]
YouTube suitable and easy to use	10 (50)	“Using YouTube was a bit cumbersome due to the login process.” [Jasmine]
Social aspects of YouTube satisfying	17 (85)	“The social aspects of YouTube were satisfying, though I wished for more interaction options.” [Keisha]
YouTube website helpful	16 (80)	“YouTube was helpful for accessing the videos, but more variety would have been nice.” [Keisha]
Yoga instructor suitable	15 (75)	“The yoga instructor was excellent and made the sessions engaging.” [Shanice]
Physical trainer suitable	14 (70)	“The physical trainer provided clear instructions that were easy to follow.” [Tanisha]
Chair Yoga Program satisfying	14 (70)	“The Chair Yoga Program was satisfying and perfect for my needs.” [Jasmine]
Floor Mat Program satisfying	15 (75)	“I enjoyed the Floor Mat Program. It was a great workout.” [Ebony]
Participation in study satisfying	19 (95)	“Overall, participating in this study was a very satisfying experience.” [Jasmine]

^a^BP: blood pressure.

### Benefits of and Barriers to YogicDance Intervention

Of the 22 study completers, 17 attended the postintervention, semistructured focus group interviews. The following 4 important themes emerged from the qualitative analyses: accountability, increased awareness, health benefits, and unanticipated stressors (Table 5).

**Table 5:** Themes and subthemes derived from YogicDance qualitative data (n=17).

Theme	Related word frequency	Subtheme	Exemplar quotes
Accountability	Accountability (n=14), accountable (n=11)	Black women feel they need to be accountable to others before themselves; they put themselves last. Study participation provided opportunities for self-accountability via self-monitoring devices; however, there were missed opportunities for accountability via social support. The social support aspect of practicing yoga is missed in the home-based, video watching experience.	“...and you’re always putting everyone else first and making sure the house is standing the way that it’s supposed to be. ...we’re neglecting ourselves and it’s easier for me to tell you ‘girl if you don’t take care of yourself…’ and if my daughter sneezes the wrong way it’s like we’ve gotta make a doctor’s appointment or if my husband’s leg is bothering [him] we gotta get you to the doctor. All the while, I’m probably the sickest one in the house...” [Ebony] “...as far as accountability, I like that it’s [the intervention], a situation that I’m obligated to and I have to do it despite whatever else I have going on, so when I heard about Black women, health, [I thought] I’m there.” [Jasmine] “I need accountability in a group in regards to eating healthy and working out. I tried it by myself...but when it comes to trying to eat right and working out I can’t do what I want. So I need people that will be able to encourage me and work together towards that type of goal.” [Tamika]
Increased awareness	Awareness (n=9), aware (n=5)	Tailored health education videos provided informative, enlightening explanations regarding cardiometabolic health and safe yoga practice. Self-monitoring technology revealed negative health patterns and informed health-promoting behaviors.	“I fairly enjoyed the educational videos, surprisingly. They were very informative. Sometimes when you are presented with medical or scientific information it can be quite overwhelming but it was all presented in very layman terms.” [Aisha] “I wanted to participate in the yoga study which actually explains yoga because I needed to learn how to relax, de-stress, meditate...” [Jasmine] “I was used to being aware of where my blood pressure is, but taking it daily gave me a really good baseline of what I should be looking for; so it was extremely helpful.” [Jasmine] “...I really enjoyed it because it encouraged me to walk more, like if I took my daughter to the park, I was encouraged to walk around while she was playing instead of just sitting there. Or even at work it encouraged me to take steps...” [Monique]
Health benefits	Beneficial (n=10), benefits (n=8), benefit (n=7)	Study engagement was perceived to be a step toward lowering risk of high blood pressure and diabetes, especially in those with family history. Home-based videos and self-monitoring devices and logs provided opportunities for participants to see the immediate positive impact of intervention activities on health outcomes.	“For me it was definitely a wake-up of how I need to make time for myself. Um and the ten-fifteen minutes or the fifteen to twenty the variety um of the videos that were offered really made me think about how much time I waste not taking care of myself. Um fifteen minutes you’ll sit and watch a movie for an hour two three you’ll sit there binge on something for hours and not take care of yourself so for me it was a lot of introspection that any amount of activity of any period of day you can take the time to do for yourself...” [Shanice] “The breathing exercises were awesome for that. I didn’t know how much that would… I mean I heard people say take deep breaths and stuff like that but actually focusing on my breathing and focusing on a specific way to do it as the instructor taught us. I really had to focus on that and it took my mind off of other things. Also the breathing itself helped to just relax my mind and body. It was a new experience to learn how effective just breathing could be in reducing stress. Before, I never paid much attention to how I was breathing, but this study taught me the importance of mindful breathing and how it can significantly impact my mental state and overall well-being.” [Ebony] “The reason why I wanted to be a part of this study is because I do currently have high blood pressure...I’m trying to figure out how to reduce that and I was really hoping that I would learn some tricks and tips from the yoga. I do have a family history of high blood pressure, diabetes, and I don’t want to go too much further down that road.” [Jasmine] “I have a family history of high blood pressure, diabetes, and heart disease. And I also have an interest in yoga.” [Ebony] “It was really beneficial to me to understand my blood pressure. Since within the last year and a half I was recently diagnosed with high blood pressure. It was interesting to me to see the change of my blood pressure based on exercise, to see how I was feeling before and after...” [Aisha] “And I loved having to do my blood pressure a couple of times because I could see the impact that even walking for twenty minutes or thirty minutes a day was influencing it. So it helped to be more aware of the steps that I have to take on a consistent basis so that I don’t have to take any kind of medicine.” [Keisha]
Unanticipated stressors	Stress (n=6), stressed (n=8), stressful (n=9), distress (n=8), stresses (n=8), stressors (n=9)	Work, family, and community-level stressors influenced study engagement. YouTube could have been used for social support. Videos were time-consuming and could have had more variety.	“...I think the added stress that sometimes Black women and Black people have is that societal pressure that comes from things that happen to our community...when I was taking this survey, it was right after Philando Castile and Alton Sterling happened. And when you work with people of other races, it’s really difficult for them to understand how someone you never met, their death impacts you in a very personal way. And I was stressed when I took that survey.” [Keisha] “...and I got to a point where-I have my normal everyday stresses, and I’m doing what I can for the Black community, and there’s no more that I can do so I’m just going to go ahead and turn the TV off. That combined with Donald Trump running for president, I stopped watching the news.” [Monique] “In reference to the YouTube video website, it was kind of cumbersome. ...if I had time to go back and do an additional video or so to go back and look through the links to see which one I wanted. So say if I watched the informational video to go back or for the resistance I had to go back into the emails that I had and find them. So that was a little cumbersome. But again, I understand because of privacy.” [Jasmine] “I think YouTube is good because I think so many people use YouTube and it's very accessible. But I think again because it was the same 4 videos it was really hard to be like ‘you go, girl!’ But if it was more like ‘I just did that one,’ or ‘I’m on this one’ or if it got harder then I probably would have wanted some social support.” [Jasmine] “I just think if it was more videos it might have taken off with that piece.” [Tanisha] “I’ve mentioned this before and it’s been said, after I went through all the breathing, the endurance, the chair exercise, the floor exercise, then I was looking for something to be different the next week. But you were like oh we just recycle those and I went back and sort of got stuck in the chair exercise after that.” [Latoya]

### Accountability

Self- and group accountability for participating in health-promoting behaviors were seen by the women as both a benefit and a barrier to YogicDance engagement. The women identified with being accountable to many others in their lives, often putting themselves and their health last in priority and not being accountable to their own health promotion. Conversely, participating in a research study targeting Black women was seen as a way of holding themselves accountable for their own health and providing them opportunities for group accountability. Participation in the focus group gave them an opportunity to express the phenomenon of putting the needs of others before their own. Participants were interested in components of the intervention that encouraged them to hold themselves accountable for healthy behaviors. The women highlighted their desire to be involved in group physical activities like yoga, hoping for opportunities to be accountable to a group with similar health goals. 

### Increased Awareness

Study participants considered the intervention videos to be educational and enlightening. The women highlighted their desires to learn more about their own health in a way that was straightforward and easy to understand. They also described the benefits of increased health awareness. The study prompted self-reflection on personal health habits and lifestyle choices.

Participants became more aware of their need to prioritize self-care and manage stress effectively.

In addition to the videos, the women found the physical activity and blood pressure self-monitoring devices helpful for revealing their own negative health patterns (ie, low activity levels and elevated blood pressures). Daily self-monitoring increased awareness of these unhealthful patterns and provided real-time impetus for positive health behavior change.

### Health Benefits

Participants provided positive feedback on the yoga and breathing exercises, with many finding them relaxing and beneficial. Breathing exercises and yoga practices were highlighted as beneficial for reducing stress and improving mental clarity. Several participants mentioned the mental health benefits of focusing on breathing and relaxation techniques. The women considered engagement in the yoga intervention as a means for addressing their family histories of cardiometabolic disease. They believed, by engaging with the yoga intervention, their health would improve. Women were interested in learning more about the benefits of yoga and in experimenting with the positive health impacts of yoga practice. Participants’ tracking of blood pressures following daily YogicDance practice provided opportunities for immediate gratification, as women often noted immediate beneficial impacts on their own blood pressures following yoga videos.

### Unanticipated Stressors

Participants noted difficulties in maintaining daily routines and adhering to the study requirements due to various life stressors. Despite these challenges, many participants expressed a sense of obligation to complete the study and not let the group down. Work and community-level stressors played a role in the participants’ study engagement. The participants resonated with having a sense of personal responsibility for their local Black communities, and they identified with psychological archetypes of being strong for and supportive of their communities in general. As a result, perceived responsibilities to their communities were woven into the personal lives of the participants. The women provided examples of the stressors that resulted from their feelings of responsibility to others on the community level and the ways these feelings impacted their engagement with the YogicDance intervention. Participants exemplified stressors that emerged during the intervention, which took place during the summer before a US presidential election. They described community-level stressors related to escalating news reports of community violence and political upheaval.

Other unanticipated stressors participants encountered during the intervention had to do with their restricted use of YouTube. Prior to beginning the intervention, participants were asked to use researcher-provided usernames and login passwords to pre-assigned email accounts with affiliated YouTube accounts. They were asked to login using YouTube video links emailed to their newly assigned email accounts and not their own personal YouTube accounts. These measures were taken to protect participant identities and to preserve their rights to privacy and confidentiality. Some participants struggled with certain exercises, particularly those involving resistance bands, and suggested the need for more tailored modifications. When participants were encouraged to share their thoughts on the effectiveness of the YouTube platform itself, they provided meaningful feedback regarding their desire for the intervention to have more variety than the few restorative mat and chair yoga programs being offered. Additionally, the participants wanted more opportunities to show social support to one another. Participants provided mixed feedback on the educational videos: Some found them informative and well presented, while others felt they were too long and sometimes difficult to engage with. Suggestions for improvement included shorter, more varied videos and more interactive content.

## Discussion

### Benefits to Engagement

Our study highlights the successful implementation and positive outcomes of the YogicDance intervention, demonstrating the feasibility and acceptability among Black women at high risk for metabolic syndrome. The interpretation of the quantitative and qualitative data collected and analyzed from this explanatory sequential mixed methods study determines the quantitative results that require further explanation. Key findings include significant engagement with the intervention’s components, positive feedback on the technological aspects used for delivering the intervention, and overall satisfaction with the program’s content and delivery method, with a significant majority of participants completing the intervention and the associated acceptability surveys and focus groups. Quantitative data showed high levels of engagement, with 95% of participants completing the postintervention acceptability survey. Qualitative data supported these findings, as participants expressed appreciation for the mental health benefits of focusing on breathing and relaxation techniques; the structured, culturally tailored health education; and the ease of use of digital platforms, which facilitated engagement. These results underscore the potential of culturally tailored, internet-delivered, yoga-based interventions to support lifestyle changes and address metabolic syndrome risk factors in this population [13].

### Recruitment and Engagement Strategies

Our recruitment strategies leveraged digital platforms, which proved effective at engaging Black women in health interventions, reflecting participants’ preferences for online interactions. This preference aligns with prior research indicating the effectiveness of web-based strategies at engaging Black women in health interventions [33]. Participants appreciated the convenience and accessibility of online recruitment, which included Facebook and a university-wide e-newsletter, contributing to the successful enrollment and retention of participants. A trial using Facebook and text messages with culturally relevant content increased participation, social support, and self-reported physical activity in a study on physical activity differences [34]. The use of culturally relevant images and messaging, along with the convenience and cost-saving aspects of online recruitment, contributed to the successful enrollment and retention of participants in an internet-based survey study [35].

### Technological Engagement

Technological engagement was a critical component of the study, with participants actively using web-based surveys, instructional videos, Fitbit trackers, and electronic blood pressure monitors. Participants highlighted the benefits of digital engagement tools, such as instructional videos and Fitbit trackers, which provided a sense of accountability and motivation. The Fitbit and blood pressure monitor were generally well received for their ability to provide real-time health data. Issues with the accuracy and usability of these devices were noted, indicating a need for better onboarding and support. Quantitative data showed that 95% of participants found the web-based surveys and Fitbit trackers easy to use, underscoring the effectiveness of digital methods for engaging participants. The integration of these self-monitoring tools facilitated engagement and provided valuable insights into participants’ health behaviors. Research demonstrates the effectiveness of web-based surveys for engaging participants and providing unique insights [35], particularly in cancer screening studies where online participants were more likely to follow screening recommendations [36]. Additionally, online survey completers older than 50 years typically had higher education and income [37]. In a study focusing on physical activity, web-based monitoring was preferred over paper journals [37], highlighting the effectiveness of digital methods for engaging participants. The integration of self-monitoring tools and health education via digital platforms not only facilitated engagement but also provided insights into the participants’ health behaviors and attitudes toward managing their cardiovascular risk [38-41].

### Acceptability of YogicDance

The acceptability of the YogicDance intervention was high, with participants reporting satisfaction with the social aspects of YouTube, the instructional content, and the overall intervention. Quantitative results indicated that 95% of participants found the intervention suitable and satisfying. However, challenges with the YouTube platform’s accessibility suggest the need for more user-friendly and accessible digital tools [42,43]. Despite these challenges, the intervention’s impact on self-monitoring behaviors, particularly blood pressure monitoring, underscores the value of real-time feedback for motivating health behavior change and managing cardiovascular risk [44,45].

### Barriers to Engagement

Despite these positive findings, participants identified several barriers to engagement, including lifestyle stressors and the complexity of accessing certain technological features. Many participants faced challenges such as time management issues, family obligations, and personal health problems that interfered with their ability to fully participate. Technical issues, such as problems with the blood pressure monitor and Fitbit, also posed barriers. Some participants found the online format less engaging and struggled with self-motivation. Existing research highlights the importance of addressing barriers like stress and time constraints due to family and caretaking responsibilities in efficacious physical activity diabetes prevention lifestyle interventions [45,46]. Participants’ feedback indicated that issues with the YouTube platform’s navigation impeded their ability to engage fully and suggested a need for improved usability and additional social support features. This feedback suggests the need for interventions to better use social media’s features to enhance social support and engagement. Culturally competent care for minority women emphasizes the role of social context in health decisions, including the use of mind-body therapies [13].

### Recommendations for Future Studies

Previous research has shown the significance of social support in encouraging participation in physical activities like YogicDance [47]. Barriers such as family-related stress and lack of social support, especially among low-income Black women, underscore the need for increased social support to mitigate cardiovascular disease risks [48]. Although the health education videos and YouTube platform were found helpful, participants recommended improvements like providing all educational content upfront, breaking videos into shorter segments, and offering more flexibility in how participants engage with the social media or social support platform features for future interventions [49,50]. More in-person or interactive online sessions were also recommended to enhance engagement and accountability. Enhancing social support features on platforms like YouTube could foster a sense of community and accountability, particularly beneficial for Black women’s health [51,52]. Specifically, enhancing social support features and simplifying mobile technology use could further improve engagement and outcomes [53]. Additional recommendations were made for better onboarding for technological tools and clearer instructions for exercises. Participants also suggested incorporating more varied and advanced routines to cater to different fitness levels. These insights are crucial for refining future interventions to better meet the needs and preferences of Black women at high cardiovascular risk.

### Limitations

A key limitation of our study is the potential for selection bias, as consistent, at-home internet access was an intervention prerequisite. This may have resulted in a sample with higher income stability and better broadband access than lower-income populations. Additionally, the higher education levels of our participants may limit the generalizability of our findings to women of varying socioeconomic backgrounds.

### Conclusion

Our mixed methods study demonstrates the potential of the YogicDance intervention to engage Black women in physical activity and self-monitoring behaviors aimed at reducing cardiovascular risk. Using an explanatory sequential design approach, we gathered preliminary effect sizes for critical quantitative health biomarkers such as blood pressure, fasting blood glucose, waist circumference, and blood lipids, contributing to the groundwork for future research in this area. The qualitative insights from focus groups provide a deeper understanding of the unique benefits and barriers faced by participants, emphasizing the need for culturally tailored, user-friendly, digital tools to enhance engagement. Future research should build on these findings, addressing identified barriers such as lifestyle stressors and technical issues and leveraging digital platforms’ strengths to improve health outcomes for this high-risk population.

## Appendix

Multimedia Appendix 1 YogicDance study components. This image depicts the key components of the YogicDance study, which aimed to engage African-American/Black women at high cardiovascular risk. The study included internet-based recruitment, community-based engagement, use of activity trackers, health monitoring, health education, and internet-based surveys. These components were designed to enhance engagement, monitor health, and educate participants to reduce cardiometabolic risks.

Multimedia Appendix 2 YogicDance Facebook recruitment flier. This image displays the YogicDance research recruitment flier used on a clinical trials Facebook page of an urban university. The flier targets African-American women, featuring models from the study's demographic. It details the study's focus on beginner-level chair yoga, mat yoga, and light resistance training, and provides contact information for interested participants. The recruitment was conducted from summer 2015 to fall 2016, aiming to engage the community in a home-based research study.

Multimedia Appendix 3 Documented Extent of AI Involvement in this Manuscript.

## Abbreviations

CHERRIES: Checklist for Reporting Results of Internet E-Surveys
HPM: health promotion model
IPAQ: International Physical Activity Questionnaire
IRB: institutional review board
PAR-Q+: Physical Activity Readiness Questionnaire+
PI: principal investigator
REDCap: Research Electronic Data Capture

## Data Availability

The data sets generated or analyzed during this study are presented in the main manuscript. Additional supporting files, which include de-identified data, are available upon reasonable request. Researchers interested in accessing these files should contact the corresponding author, Dr. Candace Johnson, at cjohnso11@ltu.edu or cmc8k@virginia.edu, providing a brief description of the intended use of the data. Access will be granted to qualified researchers, ensuring adherence to ethical guidelines and data privacy regulations.
