# Supplementary figures and images for: Online Yoga Pilot Intervention for Black Women at High Cardiovascular Risk: Internet-Based Recruitment and Engagement
Source: JMIR Form Res. 2025 Sep 17;9:e41221. doi: 10.2196/41221 (PMC12489401; doi:10.2196/41221)

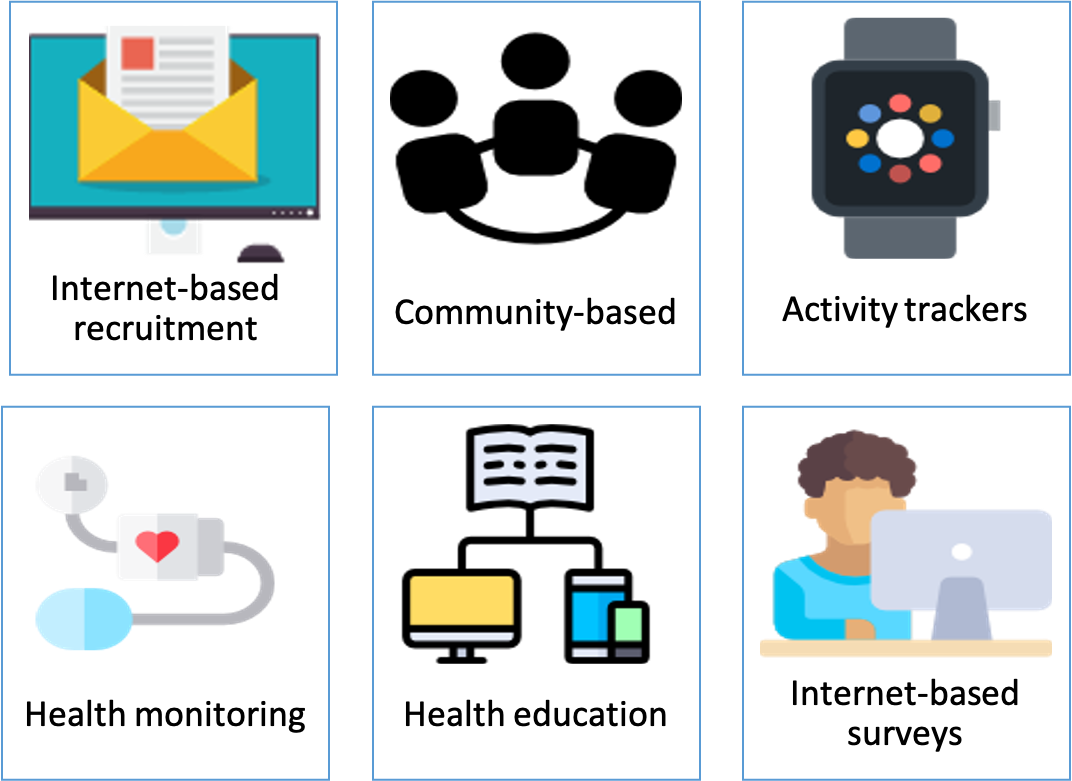

Supplement: Multimedia Appendix 1 [file formative_v9i1e41221_app1.png]

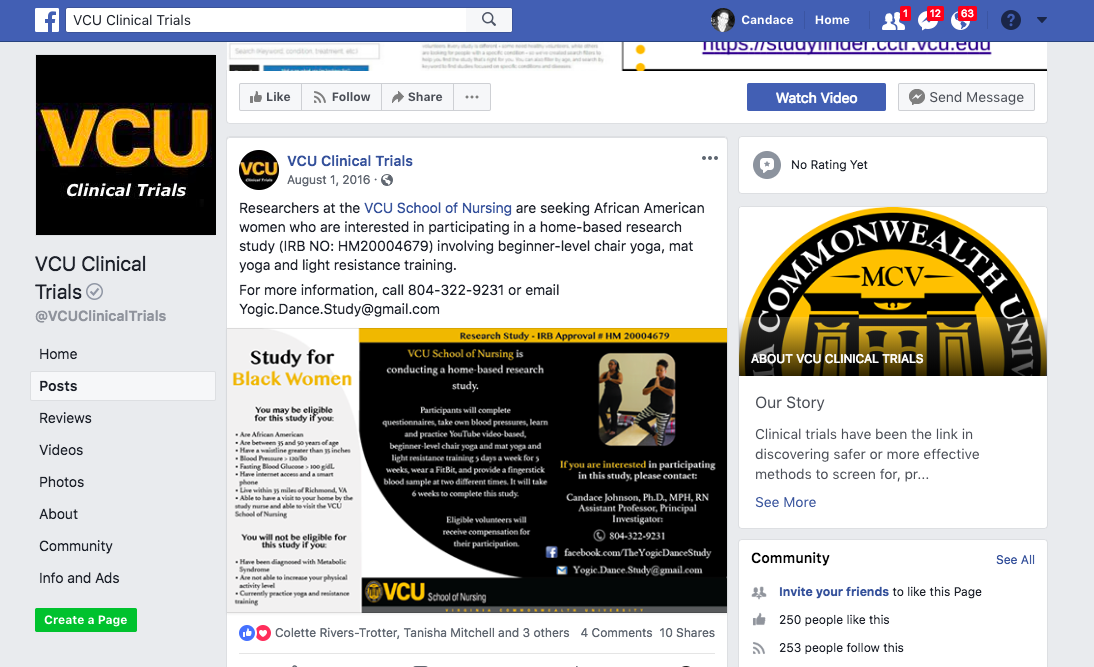

Supplement: Multimedia Appendix 2 [file formative_v9i1e41221_app2.png]
