# Supplementary material for: Online Yoga Pilot Intervention for Black Women at High Cardiovascular Risk: Internet-Based Recruitment and Engagement
Source: JMIR Form Res. 2025 Sep 17;9:e41221. doi: 10.2196/41221 (PMC12489401; doi:10.2196/41221)
Supplement: Multimedia Appendix 3 [file formative_v9i1e41221_app3.docx]

**Documented Extent of AI Involvement**

In the revision process of this manuscript, OpenAI's ChatGPT was utilized in the following ways:

1. **Addressing Reviewer Comments**:

- Assisted in generating structured responses to each of Reviewer B's comments, including reorganization of the methods section, addition of ethical considerations for focus groups, correction of the study design, and clarification of participant details.

- Assisted in revising Figure 4 and integrating quantitative and qualitative findings.

2. **Text Generation for Revisions:**

- Proofread and revised new text for the "Ethical Considerations" section to include handling of focus group data.

- Proofread and revised text for the "Qualitative Results" section by adding participant numbers to quotes.

- Proofread and revised a new section on the integration of quantitative and qualitative findings.

3. **Enhancing Clarity and Coherence:**

- Assisted in rephrasing and refining manuscript content to improve clarity and readability.

- Ensured a logical flow and organization in the revised manuscript sections.

4. **Compliance and Documentation:**

- Advised on compliance with JMIR's AI usage policy.

- Ensured transparency by documenting the AI's involvement and retaining transcripts of interactions for submission as supplementary materials.

This documented extent highlights the specific areas where ChatGPT contributed to the manuscript revisions, ensuring a transparent and ethical use of AI in the academic publishing process.
